# A Comprehensive Analysis of Single-Incision Laparoscopic Cholecystectomy: Trends, Challenges, and Future Directions

**DOI:** 10.7759/cureus.54493

**Published:** 2024-02-19

**Authors:** Rami Kamal Atiya Morcos, Sergio Rodrigo Oliveira Souza Lima, Syed Faqeer Hussain Bokhari, Mohammed Khaleel I.KH. Almadhoun, Mitwa Patel, Nay Phone Hlyan

**Affiliations:** 1 General Surgery, Ministry of Health Holdings, Riyadh, SAU; 2 General Surgery, Ain Shams University Hospitals, Cairo, EGY; 3 Plastic Surgery, Bahia Hospital, Salvador, BRA; 4 Surgery, King Edward Medical University, Lahore, PAK; 5 Medicine and Surgery, Mutah University, Karak, JOR; 6 Medicine, David Tvildiani Medical University, Tbilisi, GEO; 7 Emergency Medicine, Sheffield Teaching Hospital National Health Service (NHS) Foundation Trust, Sheffield, GBR

**Keywords:** review, silc, sils (single incision laparoscopic surgery), laporoscopic cholecystectomy, single-incision laparoscopic cholecystectomy, minimally invasive laparoscopy

## Abstract

Single-incision laparoscopic cholecystectomy (SILC) is a minimally invasive surgical technique introduced as an advancement to laparoscopic cholecystectomy (LC). This narrative review delves into the emergence of SILC, emphasizing its distinct advantages such as improved cosmesis, reduced postoperative pain, and potentially faster recovery compared to traditional LC. The study meticulously examines current trends and challenges in SILC, including variations in techniques and their impact on patient outcomes. Furthermore, the article sheds light on the technical intricacies and longer operative times associated with SILC. It aims to contribute valuable insights to the medical community by synthesizing existing literature and recent research findings, fostering a deeper understanding of SILC, and guiding future advancements in minimally invasive surgical approaches. The discussion extends to the learning curve, complications, and a comparative analysis between SILC and traditional LC, offering a nuanced understanding of their respective strengths and limitations. The article concludes with a forward-looking perspective, exploring future directions and innovations in SILC, including advancements in surgical techniques and the integration of innovative technologies, such as robotic assistance and in vivo robots, to enhance precision and efficacy. The call for continued research into the long-term outcomes, safety, and refined patient selection criteria emphasizes the evolving landscape of SILC and its potential to shape the future of minimally invasive abdominal surgeries.

## Introduction and background

Laparoscopic cholecystectomy (LC), introduced in the late 1980s, revolutionized the field of surgery by offering a minimally invasive alternative to traditional open procedures [1]. This technique, involving small incisions and a camera-equipped laparoscope, significantly reduced patient recovery time and postoperative complications. A noteworthy milestone was the incorporation of laparoscopic cholecystectomy into general surgery within just two to three years of its introduction [2]. Single-incision laparoscopic cholecystectomy (SILC) emerged as a further advancement, refining the laparoscopic approach by utilizing a single port for access. SILC involves performing the procedure through a single incision, often hidden in the navel, resulting in enhanced cosmetic outcomes [3]. The surgeon employs specialized instruments and a flexible endoscope to navigate and perform the surgery. SILC offers distinct advantages, such as improved cosmesis, reduced postoperative pain, and potentially faster recovery compared to traditional LC [4,5].

Exploring the current trends and challenges in SILC is crucial in the context of ongoing advancements in surgical techniques. Surgeons are continually refining their approaches to enhance patient outcomes, minimize complications, and improve overall surgical efficiency. Current trends encompass exploring variations in SILC techniques, such as three-port SILS, and evaluating their impact on patient outcomes and recovery [4]. The challenges lie in addressing the technical intricacies associated with SILC, as the procedure is inherently more demanding than traditional LC, resulting in longer operative times [6]. The objective of this narrative review is to provide a comprehensive analysis of the current trends and challenges in SC. By synthesizing existing literature and recent research findings, the review aims to offer insights into the evolving landscape of SILC techniques, their impact on patient outcomes, and the hurdles faced by surgeons.

Ultimately, the goal is to contribute valuable information to the medical community, foster a deeper understanding of SILC, and guide future advancements in minimally invasive surgical approaches. The materials and methods for this narrative review encompassed a systematic search of academic databases, including PubMed, Google Scholar, Scopus, and Web of Science, conducted from inception to December 2023, using keywords related to "single-incision laparoscopic cholecystectomy (SILC), laparoscopic cholecystectomy, minimally invasive surgery, surgical techniques, outcomes, advancements, and comparisons with traditional laparoscopic procedures". Studies meeting inclusion criteria, which focused on SILC techniques, outcomes, complications, comparisons with traditional laparoscopic cholecystectomy, or patient experiences, were selected for data extraction and synthesis. Quantitative data were analyzed descriptively, while qualitative data were synthesized thematically. Ethical approval was not required as the review involved the synthesis of existing literature without human subjects or experimental interventions. Limitations included potential biases introduced by study selection and variations in methodologies, as well as publication bias inherent to narrative reviews. Overall, these methods aimed to systematically gather, analyze, and synthesize existing literature to provide insights into SILC and guide future research directions.

## Review

Single-incision laparoscopic surgery

**Figure 1:**
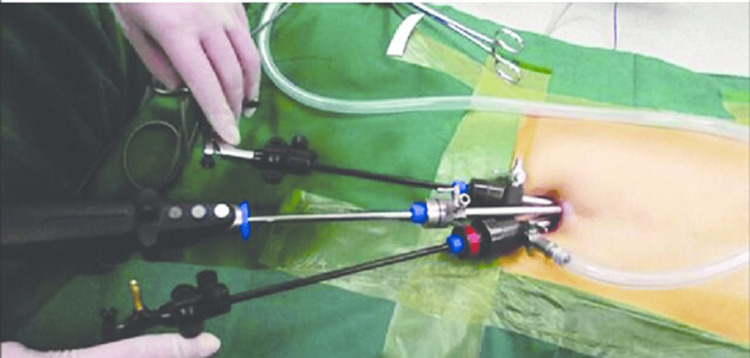
Placement of instruments in a single incision laparoscopic cholecystectomy procedure Triangular placement of instruments in single incision laparoscopic cholecystectomy showing three conventional trocars in a semicircular incision (≤ 2.5 cm in all patients) on the slope of the umbilicus. Adapted from Guo et al. [8] with permission.

Single-incision laparoscopic surgery, also known as SILS or single-port surgery, is a minimally invasive surgical technique where the entire procedure is performed through a single incision, typically made in the patient's navel. Unlike traditional laparoscopic surgeries that involve multiple incisions, SILS aims to minimize scarring and improve cosmetic outcomes while maintaining the efficacy of the surgical intervention [3,7]. The principles underlying SILS involve the use of specialized instruments and flexible scopes that allow surgeons to navigate and perform the surgery through a single entry point (Figure 1).

The technique requires advanced skills and training to manipulate the instruments within a confined space, ensuring precision and safety throughout the procedure [9]. SILS aims to replicate the outcomes of traditional LC while offering improved aesthetics [3,7]. SILS presents several advantages and potential benefits for cholecystectomy. One of the primary advantages is enhanced cosmesis due to reduced scarring, making it an appealing option for patients concerned about the aesthetic outcomes of surgery. Additionally, studies suggest that SILS may result in lower postoperative pain scores compared to traditional laparoscopy, potentially leading to a quicker recovery and a reduced need for analgesics [10,11].

Current trends in SILC

Single-incision laparoscopic cholecystectomy (SILC) has witnessed dynamic trends in recent years, transforming the landscape of minimally invasive procedures. Numerous studies and advancements have contributed to its evolution, influencing surgical techniques, patient outcomes, and the incorporation of innovative technologies. Recent studies, such as those by Chuang et al. and Gaillard et al., have extensively explored and documented the feasibility and advantages of SILC [12,13]. The focus has been on refining techniques, with SILC becoming a frequently studied and applied approach. Advancements in high-fidelity models, as discussed by Kwasnicki et al., have contributed to the improved training and implementation of SILC in surgical practice [14].

Furthermore, Rivas et al. have pioneered a two-trocar SILC technique, showcasing the continuous innovation in this field [15]. The literature also provides various definitions, such as single-incision laparoscopic surgery (SILS), single-port access, and single-laparoscopic incision transabdominal (SLIT) surgery, highlighting the diverse approaches within the SILS framework [16]. Assessing outcomes and patient experiences is integral to understanding the effectiveness of SILC. A study by Yilmaz et al. emphasizes the importance of patient experiences and satisfaction in the context of SILC [16]. Evaluating postoperative pain, recovery times, and cosmetic outcomes has become crucial in determining the success of SILC procedures.

Research has consistently shown that SILC is associated with reduced postoperative pain and shorter recovery periods compared to traditional laparoscopic approaches. Rivas et al. reported findings supporting the feasibility and reproducibility of SILC, suggesting its potential benefits for patient recovery [15]. The exploration of innovative technologies and instruments has been a driving force in advancing SILC. Kwasnicki et al. highlight the use of high fidelity models, allowing surgeons to enhance their skills and adapt to the intricacies of single-incision procedures [14]. The integration of advanced instruments has facilitated the application of SILS in various abdominal surgeries beyond cholecystectomy. Moreover, the continuous refinement of instruments has contributed to improved ergonomics and precision in SILC. These developments aim to overcome the technical challenges associated with reduced incisions while ensuring optimal surgical outcomes. The incorporation of robotic assistance is also an area of exploration, with ongoing studies assessing its role in enhancing the capabilities of SILC procedures.

In conclusion, the current trends in SILC reflect a dynamic landscape shaped by ongoing studies, advancements in surgical techniques, and the integration of innovative technologies and instruments. Evaluation of patient outcomes and experiences remains a central focus, emphasizing the importance of refining and optimizing SILC procedures for enhanced surgical outcomes and patient satisfaction. As technology continues to evolve, SILC is poised to play a significant role in the future of minimally invasive abdominal surgeries.

Challenges in implementing SILC

Implementing SILC poses significant technical challenges for surgeons. Greaves and Nicholson emphasize the twofold nature of these challenges: the inherent technical complexity of the procedure and the need for surgeons to adapt to it willingly and proficiently [9]. The single incision demands a high level of precision and dexterity, requiring surgeons to navigate limited space while maintaining the same standards of safety and efficacy as traditional laparoscopic procedures. Learning the novel techniques associated with SILC presents a substantial hurdle. Kurpiewski et al. highlight the learning curve associated with SILC, indicating that surgeons may face challenges during the initial stages of adopting this approach [6]. Mastering the required skills is crucial for ensuring optimal patient outcomes and avoiding complications.

The learning curve is a critical aspect of adopting SILC. Pietrabissa et al. discuss the challenges and successes associated with robotic single-site cholecystectomy, emphasizing the importance of achieving high standards of safety and efficacy through continuous learning and adaptation [17]. Surgeons need time to familiarize themselves with the unique aspects of SILC, such as the use of specialized instruments and the adjustment to a single access point. The complexity of the learning curve can impact the overall efficiency of implementing SILC in routine surgical practice. Learning curves can vary among surgeons, further complicating the widespread adoption of SILC. Culp et al. suggest that the slow beginning of the learning curve is associated with increased rates of conversions and complications, underscoring the need for surgeons to invest time and effort in becoming proficient with SILC techniques [3]. The challenges posed by the learning curve necessitate a strategic approach to training and mentorship to ensure a smooth transition for surgeons incorporating SILC into their repertoire.

Complications in SILC are not uncommon and require careful consideration. Valverde et al. discuss inherent difficulties in the procedure, including the need to abandon certain established principles of LC, which may contribute to complications [18]. Complications can range from bile duct injuries and surgical site infections to issues such as seroma and wound pain, as reported by a study analyzing 150 consecutive patients undergoing single-port LC [19]. These complications underscore the importance of proper training, surgeon experience, and patient selection in minimizing adverse events associated with SILC. Limitations in instrumentation, particularly in the context of a single incision, can contribute to technical challenges and increase the risk of complications. Surgeons must navigate these limitations while maintaining a commitment to patient safety and positive outcomes.

Comparison of SILC and traditional LC

**Figure 2:**
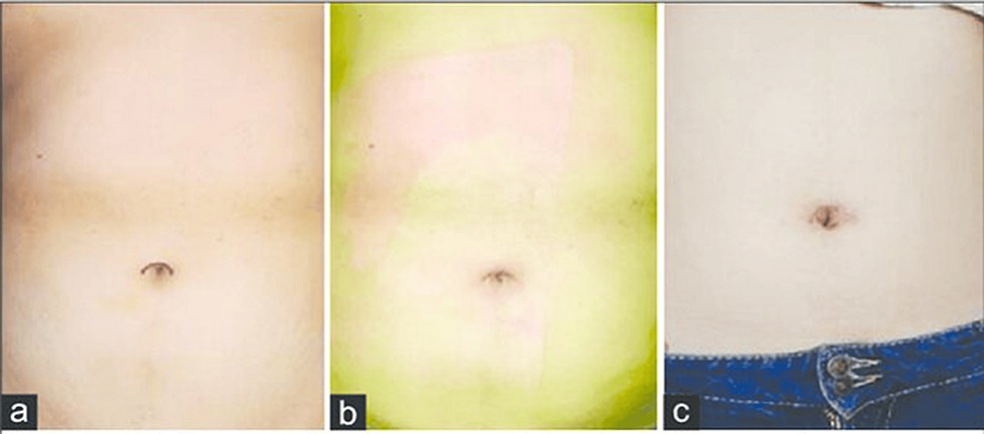
Incision in single‑incision laparoscopic cholecystectomy (a) shows the mark of the incision site, (b) and (c) shows a curved scar present 7 days and 30 days after surgery, respectively. Adapted from Guo et al. [8] with permission.

A comparison of SILC with traditional laparoscopic approaches reveals a growing body of evidence assessing the benefits and limitations of these surgical techniques. In a study by Hajong et al., SLIC was found to be a safe and feasible procedure comparable to multi-incision laparoscopic cholecystectomy (MILC) [20]. This study emphasizes the need for a step-by-step approach to ensure the success of SILC, indicating that the technique offers comparable outcomes to traditional multi-incision approaches. Numerous studies, including a meta-analysis conducted by Geng et al., have delved into the outcomes, safety, and efficacy of SLIC. The meta-analysis revealed that SILC offered a better cosmetic result and less postoperative pain for patients with uncomplicated cholelithiasis or polypoid lesions [21]. It also highlighted the longer operative time and additional instrumentation associated with this procedure. These finding aligns with the increasing interest in SLIC as a minimally invasive technique that not only addresses medical concerns but also considers the aesthetic aspect of patient satisfaction (Figure 2).

A systematic review with a meta-analysis conducted by Evers et al. further assessed safety, patient-reported outcome measures, and the feasibility of SILC compared to conventional laparoscopic approaches [22]. The analysis provides valuable insights into the overall performance of SILC, contributing to the ongoing dialogue surrounding its adoption in surgical practice.

The success of SILC is intricately tied to patient selection criteria. Gupta et al. discuss the comparison of cosmetic outcomes between SILC and conventional LC, emphasizing the need to consider demographic and clinical factors when opting for SILC [23]. Factors such as body mass index, the presence of comorbidities, and the complexity of gallbladder disease play a pivotal role in determining the suitability of patients for SILC. Karim et al. conducted a study comparing the outcomes of SILC to conventional multiport LC, focusing on morbidity parameters [24]. The analysis of different morbidity parameters, including complications, provides valuable information for surgeons to refine patient selection criteria, ensuring optimal outcomes and minimizing adverse events associated with SILC.

Artificial intelligence and SILC

The amalgamation of artificial intelligence (AI) and SILC represents a significant stride in the realm of modern surgical interventions. This convergence holds the potential to enhance the precision, safety, and overall efficacy of SILC procedures. AI contributes to SILC in various facets, starting with surgical planning. The application of AI algorithms in analyzing extensive datasets enables surgeons to tailor SILC procedures according to patient-specific parameters. Furthermore, AI plays a pivotal role in predictive analytics for postoperative outcomes in SILC. Machine learning algorithms can analyze preoperative data to anticipate potential complications and guide surgeons in adopting preventive measures. This proactive approach not only enhances patient safety but also contributes to the continuous improvement of SILC protocols [25]. In parallel, the clinical landscape has witnessed an increase in the adoption of AI-driven technologies to assist in decision-making during SILC procedures. The use of AI tools provides real-time insights into patient anatomy, aiding surgeons in making informed decisions and enhancing the overall precision of the surgery [25,26]. While AI presents promising avenues for the advancement of SILC, caution is warranted. Studies have highlighted concerns regarding the higher bile duct injury rate associated with single-incision laparoscopic cholecystectomy compared to traditional approaches, emphasizing the importance of a judicious approach in implementing new technologies [27-29].

Future directions and innovations

As the field of minimally invasive surgery continues to evolve, ongoing research in SILC is exploring novel approaches and techniques. Lunevicius et al. highlight the importance of balancing potential medical and non-medical harms when introducing new techniques and technologies for LC [30]. This indicates a growing awareness of the need for comprehensive assessments that consider both medical outcomes and patient experiences. The outcomes of SILC have been a subject of interest, and Kurpiewski et al. aimed to assess the benefits to patients of applying SILC as a method of gallbladder removal. The study underlines the importance of evaluating patient-centric benefits and aligning surgical innovations with improved patient outcomes [6]. Future research could build on these findings to refine surgical approaches, ensuring a patient-centered focus in the development of new techniques.

Advancements in SILC are not only limited to surgical techniques but also extend to innovative technologies. Arroyo et al. discuss the development of SILC, laparoendoscopic single-site surgery (LESS) and the emergence of various other new techniques for gallbladder removal [7]. This points towards a trajectory where technological innovations may play a pivotal role in enhancing the precision and efficacy of SILC procedures. The exploration of miniature in vivo robots for LESS, as demonstrated by studies like Yilmaz et al. [16] and Valverde A [18] and , showcase the potential integration of robotics in the field of SILC. The use of such robots aims to enhance visualization and provide off-axis dexterous manipulation capabilities, marking a significant stride toward more advanced and intricate surgeries.

To propel the field of SILS forward, further research and development should focus on several key aspects. First and foremost, continued investigations into the long-term outcomes and safety of SILC are crucial. This involves monitoring patients over extended periods to assess the durability of the procedure and identify any potential late complications. Moreover, refining patient selection criteria is an area ripe for exploration. Gupta et al. emphasized the importance of considering demographic and clinical factors when opting for SILC, indicating that personalized approaches may enhance the success rates of the surgery [23]. Future research should delve deeper into developing robust criteria that allow for optimal patient selection, thereby maximizing the benefits of SILC.

## Conclusions

SILC, introduced as a minimally invasive alternative to traditional laparoscopic cholecystectomy (LC), offers distinct advantages such as enhanced cosmesis, reduced postoperative pain, and potentially faster recovery. The study meticulously explores current trends, emphasizing ongoing advancements in surgical techniques and the integration of innovative technologies, such as robotic assistance and in vivo robots. It sheds light on the challenges faced by surgeons in implementing SILC, including the inherent technical complexity and the associated learning curve. A comparative analysis with traditional LC reveals a nuanced understanding of the strengths and limitations of each approach, considering outcomes, safety, and patient satisfaction. The article concludes with a forward-looking perspective, advocating for continued research into long-term outcomes, safety, and refined patient selection criteria. The call for a patient-centered focus on the development of new techniques and the exploration of technological innovations, including miniature in vivo robots, highlights the potential trajectory of SILC in shaping the future of minimally invasive abdominal surgeries.
